# A Magnetic-Bead-Based Immunoassay with a Newly Developed Monoclonal Antibody for Rapid and Highly Sensitive Detection of Forchlorfenuron

**DOI:** 10.3390/bios13060593

**Published:** 2023-05-30

**Authors:** Yubao Shan, Ting He, Ying Li, Jiang Zhu, Xiali Yue, Yunhuang Yang

**Affiliations:** 1 State Key Laboratory of Magnetic Resonance and Atomic Molecular Physics, Key Laboratory of Magnetic Resonance in Biological Systems, National Center for Magnetic Resonance in Wuhan, Wuhan Institute of Physics and Mathematics, Innovation Academy for Precision Measurement Science and Technology, Chinese Academy of Sciences—Wuhan National Laboratory for Optoelectronics, Wuhan 430071, China; shanyubao@wipm.ac.cn (Y.S.); liying@apm.ac.cn (Y.L.); jiangzhu@apm.ac.cn (J.Z.); 2 Department of Chemistry, College of Science, Huazhong Agricultural University, Wuhan 430070, China; yxl@mail.hzau.edu.cn; 3 University of Chinese Academy of Sciences, Beijing 100049, China; 4 Optics Valley Laboratory, Wuhan 430074, Hubei, China

**Keywords:** forchlorfenuron, monoclonal antibody, magnetic bead, detection, food safety

## Abstract

Forchlorfenuron (CPPU) is a widely used plant growth regulator in agriculture, and CPPU residue in food can cause harm to human health. Thus, it is necessary to develop a rapid and sensitive detection method for CPPU monitoring. In this study, a new monoclonal antibody (mAb) against CPPU with high affinity was prepared by a hybridoma technique, and a magnetic bead (MB)-based analytical method was established for the determination of CPPU by a one-step procedure. Under optimized conditions, the detection limit of the MB-based immunoassay was as low as 0.0004 ng/mL, which was five times more sensitive than the traditional indirect competitive ELISA (icELISA). In addition, the detection procedure took less than 35 min, a significant improvement over the 135 min required for icELISA. The selectivity test of the MB-based assay also showed negligible cross-reactivity with five analogues. Furthermore, the accuracy of the developed assay was assessed by the analysis of spiked samples, and the results agreed well with those obtained by HPLC. The excellent analytical performance of the proposed assay suggests its great potential for routine screening of CPPU, and it provides a basis for promoting the application of more immunosensors in the quantitative detection of low concentrations of small organic molecules in food.

## 1. Introduction

Forchlorfenuron (CPPU) is a synthetic plant growth regulator with strong cytokinin-like activity [1]. It has become a popular agrochemical to boost size and improve the quality of fruit [2,3]. CPPU has been extensively used due to the growing market demand for high-quality fruits, which poses a potential health risk to consumers exposed to CPPU from food ingestion. Thus, many countries have set legal limits for the agricultural use of CPPU. For example, the maximum residue limit (MRL) for CPPU in China is set as 0.05 mg/kg in kiwifruit and grapes and 0.1 mg/kg in melon [4], while the legal limit of CPPU in the European Union (EU) is set as 0.01 mg/kg in various fruits [5]. Recently, an increasing number of studies have reported the possible toxic effects of CPPU, and the residue of CPPU in food has gradually become a concern for food safety control. A recent toxicity study of CPPU in rats revealed that CPPU has potential adverse effects on the ovaries and on the production of steroid hormones [6]. In addition, animal studies in zebrafish have revealed that CPPU can induce cardiac morphology deformation, cardiac contractile dysfunction, and erythrocyte reduction [7,8]. Given these results, it is necessary to establish analytical methods to monitor and control CPPU residue in food.

Nowadays, various instrument-based methods for CPPU detection have been developed, such as high-performance liquid chromatography (HPLC) [9], liquid chromatography–tandem mass spectrometry (LC/MS–MS) [10], and liquid chromatography time-of-flight mass spectrometry (LC/TOF–MS) [11]. However, they need well-trained technicians, extensive sample pre-treatment, and sophisticated instrumentation, which largely limits their potential for rapid screening of numerous samples. Alternatively, antibody-mediated immunoassays, such as enzyme-linked immunosorbent assay (ELISA) and nanomaterial-based strips, have been widely developed for food hazardous determination with advantages of simplicity, rapidity, cost effectiveness, and high throughput [12]. Up until now, there have only been a few immunoassay reports on CPPU, differing in their assay performance [13,14,15,16]. Antibodies serve as core reagents in immunoassays, which largely determine the specificity and sensitivity of the resulting immunodetection technique. However, there are few commercially available antibodies against CPPU, and they are costly. Abad-Fuentes’ group [17,18] produced a series of monoclonal antibodies (mAbs) and polyclonal antibodies (pAbs) with high affinity to CPPU (IC_50_ < 1 nM). Then, a direct competitive ELISA (dcELISA) based on an mAb (s5#34) was developed for CPPU analysis, showing an IC_50_ of 63 ng/L in buffer but a cross-reactivity (CR) of 71% with the herbicide thidiazuron (TDZ) [14]. Afterwards, Suarez-Pantaleon et al. [15] advocated to analyze the CPPU residues by lateral flow immunoassay (LFIA). Therefore, another mAb, P6#42, was employed to establish a LFIA for the rapid detection of CPPU, which can complete the detection in 30 min, but the detection sensitivity (IC_50_ of 286 ng/L) was significantly decreased compared with that of dcELISA (IC_50_ of 50 ng/L). Recently, a new mAb against CPPU has been produced and reported to be used to develop an indirect competitive ELISA (icELISA) [16]. However, the established icELISA exhibited an IC_50_ value of 1.04 ng/mL, and the procedure needs as long as 80 min for incubation steps, which showed less superiority over the reported dcELISA and LFIA. Considering that the toxicological profiles of CPPU and its metabolites have been intensively studied [19] as well as the increasingly stringent regulations regarding the presence of contaminants in the environment and food, it is thus of great significance to establish more sensitive and rapid quantitative methods for CPPU screening.

With the advance of nanotechnology, magnetic beads (MB) have been notably used as platform in immunosensoring and biosensing due to their unique large surface areas, biocompatibility, and magnetic behavior, as they can be used instead of a microplate as a support carrier to immobilize biometric components, and they are easily separated by an external magnet, which facilitates improved analytical sensitivity and shortened detection time [20]. At present, MB-based immunoassays and immunosensors have been employed for the detection of various analytes, e.g., aflatoxin B_1_ [21], fumonisins [22], ciguatoxins [23], and disease-relative biomarkers [24].

**Figure 1:**
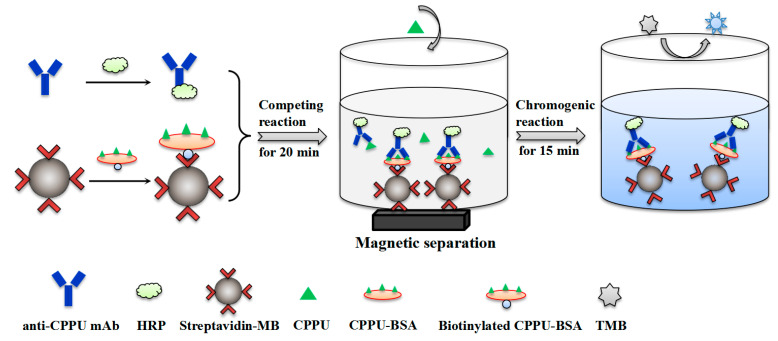
Schematic diagram of the MB-based assay.

In this work, we aim to prepare a new CPPU-specific mAb and explore the magnetic-bead-based analytical method for highly sensitive detection of CPPU in fruit samples. As a result, the specific hybridoma antibodies were isolated with high affinity against CPPU, and then the best mAb (14G1) was labeled with horseradish peroxidase (HRP) to form a tracer. MBs were employed for loading CPPU–bovine serum albumin (CPPU–BSA) to form MB–CPPU–BSA as solid-phase probe. The principle of the assay (Figure 1) is based on competition for mAb–HRP binding sites between free-CPPU and MB–CPPU–BSA. After a careful optimization of reaction conditions, the MB-based assay exhibited the desired specificity and high sensitivity for the analysis of CPPU with the detection limit as low as 0.0004 ng/mL. Meanwhile, the developed assay requires only a 20 min incubation step for antigen–antibody binding, and the sample test time does not exceed 35 min. The proposed assay can be used as a simple, sensitive, and rapid analytical tool for routine screening of CPPU in food. In combination with the advantages of magnetic beads, the established assay has great potential for further implementation into immunosensors for fast and reliable detection of small molecular agrochemicals.

## 2. Materials and Methods

### 2.1. Chemicals and Reagents

Female BALB/c mice (8 weeks old) were provided by the Experimental Animal Center of China Three Gorges University. SP2/0 myeloma cells were donated by the Cooperative Innovation Center for Sustainable Pig Production at Huazhong Agricultural University in China. Analytical grade CPPU standard (98% purity) was purchased from J&K Chemical (Beijing, China). Standards of CPPU analogues were from Yuanye Biotech Co., Ltd. (Shanghai, China). CPPU–ovalbumin conjugate (CPPU–OVA) was obtained from Shandong lvdu biotechnology Co., Ltd. (Binzhou, China). CPPU–bovine serum albumin conjugate (CPPU–BSA), goat anti-mouse immunoglobulin horseradish peroxidase (IgG–HRP), polyethylene glycol 1450 (PEG1450, 50%), and Biotin labeling kit were from Biodragon Immunotechnologies (Beijing, China). Freund’s adjuvants, hypoxanthine and thymidine (HT), and hypoxanthine/aminopterin/thymidine (HAT) were supplied by Sigma-Aldrich (St. Louis, MO, USA). Reagents for cell culture were supplied by Gibco BRL Life Technologies (Grand Island, NY, USA). Fetal bovine serum (FBS) was procured from Hangzhou Sijiqing Biological Engineering Materials Co., Ltd. (Hangzhou, China). The mouse monoclonal antibody isotyping kit and HRP conjugation kit were purchased from Proteintech (Wuhan, China). Streptavidin-modified magnetic beads were purchased from Chongqing Farsighted-Blue-Dragon (FBD) Biotechnology Co., Ltd. (Chongqing, China). Graphitized carbon black (GCB) and primary secondary amine (PSA) were procured from CNW Technologies (Shanghai, China). Cell culture plates (6, 24, and 96 wells) and 96-well polystyrene microtiter plates were from Corning (Corning, NY, USA). The 3,3′,5,5′-tetramethyl benzidine (TMB) and non-fat milk powder were obtained from Sangon Biotech Co., Ltd. (Shanghai, China). All other chemicals and agents used were of analytical grade unless otherwise specified. The measurement of optical density (OD) was conducted with the use of a SpectraMax i3x microplate reader (Molecular Devices, Sunnyvale, CA, USA). The Hitachi (Tokyo, Japan) Chromaster HPLC system consisted of an ultraviolet (UV) detector and a Symmetry C18 column (150 mm × 4.6 mm, 5 μm; Waters), and it was employed for chromatographic separations.

### 2.2. Production of Monoclonal Antibody aganist CPPU

The hapten CPPU-COOH was synthesized by introduction of alkyl mercaptoacids through the pyridine ring of CPPU [25]. The synthesis route of hapten CPPU-COOH is shown in Figure S1. The immunogen CPPU–OVA conjugate and coating antigen CPPU–BSA conjugate were obtained by the active ester method.

Female BALB/c mice were immunized four times with the CPPU–OVA conjugate. At the first immunization, we dissolved 80 μg of CPPU–OVA conjugate in 0.85% NaCl solution and emulsified with the same amount of Freund’s completed adjuvant. Mice were injected subcutaneously with the emulsion, followed by three subsequent immunizations with 80 μg of CPPU–OVA fully emulsified with Freund’s incomplete adjuvant at weeks 3, 5, and 7 after the first immunization. Antisera were obtained from the caudal vein of every mouse 10 days after each injection. These antisera were then subjected to indirect ELISA (CPPU–BSA conjugate used as coating) and indirect competitive ELISA (CPPU standard used as competitor) to test for anti-CPPU antibodies. The mouse whose antiserum exhibited higher sensitivity with CPPU was given an intraperitoneal booster 3 days before cell fusion. The booster injection used 40 μg of CPPU–OVA conjugate without adjuvant. The spleen cells (~1.0 × 10^8^) were mixed with freshly isolated SP2/0 myeloma cells (~2.5 × 10^7^) in the presence of 50% (*w*/*v*) PEG1450 for cell fusion. The cultivation procedure of hybridoma cells followed the reported study [26].

To screen the hybridoma cells, an icELISA was conducted using CPPU–BSA as the coating antigen and CPPU standard as the competitor. The positive hybridomas were subcloned three times in succession through limiting dilution to obtain monoclonal cells and ensure their stability. The selected hybridoma cells were intraperitoneally injected into BALB/c mice, which had previously been treated with 0.5 mL of Freund’s incomplete adjuvant. The mAbs were purified from ascites fluid by the caprylic acid-ammonium sulfate precipitation method. The isotype identification of the mAbs was performed using an antibody isotyping kit according to the manufacturer’s manual. The affinity of the anti-CPPU mAb was determined by indirect non-competitive ELISA [27].

### 2.3. Development of Conventional icELISA

A conventional icELISA was developed for CPPU detection as follows: 0.5 μg/mL CPPU–BSA was dropped into the 96-well microplate (100 μL/well) and incubated overnight at 4 °C. The plates were blocked at 37 °C with 5% skim milk in PBST (PBS containing 0.05% Tween 20) for 1 h. After washing the plate with PBST three times, 50 μL of mAb 14G1 and 50 μL of sequentially diluted CPPU standard solution were added. The plate was incubated for 1 h at 37 °C, washed three times, and 100 μL/well of goat anti-mouse lgG-HRP solution was added for another 1 h of incubation. After washing six times, 100 μL/well of TMB substrate was added to the plate and reacted for 15 min. Finally, 2 M H_2_SO_4_ (50 μL/well) was added into the plate to stop the reaction, and the OD_450_ value was read with a microplate reader.

### 2.4. Development of MB-Based Assay

#### 2.4.1. Immobilization of Biotinylated CPPU–BSA on Streptavidin Magnetic Beads

The streptavidin-modified magnetic beads (SA-MB) were functionalized with biotinylated CPPU–BSA conjugate as follows. First, CPPU–BSA was biotinylated according to the specification provided by the supplier. Briefly, the biotin stock solution (10 mM) was added to the CPPU–BSA solution (mass ratio was 20:1), and the mixture was gently mixed and incubated at 37 °C for 30 min away from light. After the reaction, the product was placed in an ultrafiltration tube and washed with PBS twice to remove residual biotin. The supernatant containing the biotinylated CPPU–BSA was collected after centrifuging at 12,000× *g* for 12 min. Then, 0.25 mL of 20 g/L SA-MB was added to a tube and washed with washing buffer (20 mM Na_2_HPO_4_ + 0.05% Tween 20, pH 7.4) three times. After magnetic separation, SA-MB was mixed with 0.4 mg of biotinylated CPPU–BSA, shaken gently at room temperature for 0.5 h, and washed with washing buffer three times to obtain MB–CPPU–BSA. The ratio of biotinylated CPPU–BSA to SA-MB was optimized by direct ELISA. Finally, the MB–CPPU–BSA products were suspended in 5.0 mL of PBS and stored at 4 °C for further use.

#### 2.4.2. Preparation of HRP-Labeled mAb

Anti-CPPU mAb 14G1 labeling HRP was performed according to the specification provided by the supplier (Proteintech, Wuhan, China; PK20001). In brief, 20 μL of modifier reagent was added to 200 μL of mAb 14G1 solution (0.5 mg/mL), mixed lightly, transferred to a tube containing 100 μg of HRP freeze-dried powder, mixed well, and incubated at 37 °C for 3 h. After that, 20 μL of quencher reagent was added to the mixture, thoroughly mixed, and left to stand at 37 °C for 1 h. Finally, an equal volume proportion of glycerol was added into the mAb–HRP solution and stored at −20 °C for further use.

#### 2.4.3. MB-Based Assay Procedure

A direct competitive MB-based assay was developed as follows. First, the 96-well microtiter plate was pre-sealed with 5% skim milk at 37 °C for 1 h. After washing with PBST, MB–CPPU–BSA (10 μg/μL, 10 μL per well) was added to the 96-well plate. Subsequently, 50 μL of serially diluted CPPU standards and 50 μL of diluted mAb–HRP (1:3200) were mixed into the wells. The plate was shaken well and incubated at 37 °C for 20 min and then placed on a magnetic base to precipitate the MB. Then, the supernatant was discarded, and the plate was washed three times with PBST. After washing, TMB substrate (100 μL per well) was added and incubated for 15 min at 37 °C. The reaction was stopped with 2 M H_2_SO_4_, and the absorbance was measured at a wavelength of 450 nm.

#### 2.4.4. Optimization of MB-Based Assay

The reaction parameters greatly influence assay sensitivity. Therefore, the experimental parameters including concentrations of MB–CPPU–BSA and mAb–HRP, ionic strength, pH value, and organic solvent content in buffer were investigated to improve the sensitivity of the MB-based assay. After optimization, the standard curve for the MB-based assay was generated by plotting the B/B_0_ (B and B_0_ were defined as the OD_450_ in the presence and absence of CPPU, respectively) against the logarithm of CPPU concentration. The 50% inhibitory concentration value (IC_50_) calculated from the curves was used to evaluate assay sensitivity.

### 2.5. Selectivity Determination

The cross-reactivities (CRs) of the developed MB-based assay and icELISA with compounds structurally related to CPPU were used to evaluate the selectivity of the assays. The CR value of each compound was calculated as follows: CR (%) = (IC_50_ of CPPU/IC_50_ of analyte) × 100%.

### 2.6. Sample Preparation

Kiwifruit and grape samples obtained from a local market were confirmed as CPPU-free by HPLC analysis. The negative samples were subjected to recovery tests by spiking serial concentrations of CPPU standard. The samples were pretreated according to the Chinese National Standard method with slight modifications [28]. A total of 10.0 g of each homogenized sample was precisely weighed into 50 mL tubes, and 10.0 mL of acetonitrile was added, shaken for 2 min, and subjected to an ultrasound extraction for 25 min. After that, 1.0 g of sodium chloride and 4.0 g of anhydrous sodium sulfate were added, and the tubes were shaken for 2 min and centrifuged for 5 min at 5000 rpm. The supernatants were collected, and 2.0 mL of the supernatant was filtered with a 0.22 µm organic-phase filter for MB-based assay analysis. Then, 1.0 mL of remaining supernatant was mixed with 200 mg of primary secondary amine (PSA), 200 mg of anhydrous sodium sulfate, and 2.5 mg of graphitized carbon black (GCB), and then it was shaken vigorously for 1 min and centrifuge at 5000 rpm for 5 min. The final supernatants were filtered through 0.22 µm organic-phase filters for HPLC analysis. In the HPLC system, a mobile phase consisting of HPLC-grade acetonitrile mixed with water in a 40:60 ratio was used, and the elution flow rate was 1.0 mL/min. The injection volume was 20 μL. The temperature of the column was maintained at 30 °C, and the UV detector was operated at 260 nm.

## 3. Results and Discussion

### 3.1. Production and Characterization of Anti-CPPU mAb

**Figure 2:**
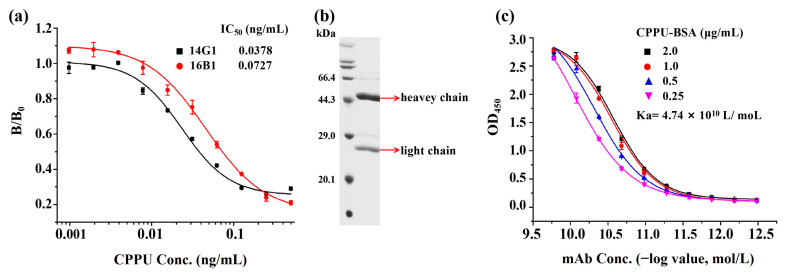
Characterization of hybridoma antibodies. (**a**) Sensitivity determination of hybridoma antibodies. (**b**) SDS-PAGE analysis of mAb 14G1 after purification from ascitic fluid. (**c**) Affinity constant determination of mAb 14G1 and CPPU.

A total of 480 hybridoma cell lines were seeded into 96-well plates. After the screening of fusion and subclones, two stable positive hybridoma clones (14G1 and 16B1) were selected. The subtypes of the two clones were identified by a commercial kit, and the results showed that both clones belonged to the IgG1 type, and the light chain isotype was kappa. Dose–response curves of clones 14G1 and 16B1 for CPPU were obtained in Figure 2a. The curve was generated by plotting the B/B_0_ versus the logarithm of the CPPU concentration. In this context, B represents the optical density of the analyte at each concentration, while B_0_ represents the optical density when the analyte is absent [29]. Clone 14G1 was selected for further detailed examination because it showed higher sensitivity in IC_50_ value (0.0378 ng/mL) than that of 16B1 (IC_50_: 0.0727 ng/mL), as shown in Figure 2a. Then, the mAb 14G1 was purified from ascitic fluid and analyzed by 15% SDS-PAGE, which exhibited two characteristic bands: heavy chain (~50 kDa) and light chain (~25 kDa) (Figure 2b). As shown in Figure 2c, the affinity constant (Ka) of the purified mAb 14G1 was calculated as 4.74 × 10^10^ L/mol, which reflected high affinity between the mAb and its corresponding antigen [30].

### 3.2. Optimization of an Indirect Competitive ELISA

**Figure 3:**
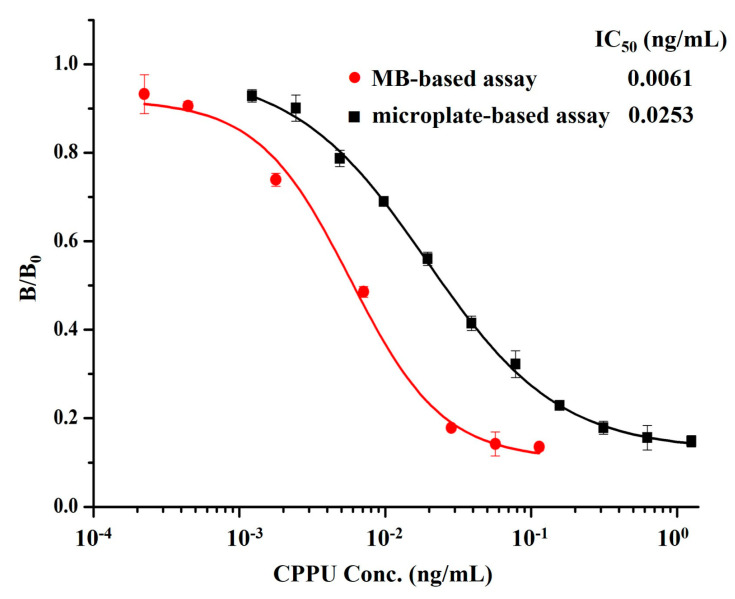
Standard inhibition curves of developed MB-based assay (●) and conventional icELISA (■) for CPPU under optimized parameters (*n* = 3).

The experimental conditions of conventional icELISA were optimized. The evaluation criteria were the 50% inhibition concentration (IC_50_; representing the sensitivity) and maximum absorbance (OD_max_) of the negative control. The working concentrations of the CPPU–BSA conjugate and mAb 14G1 were primarily optimized by a checkerboard titration. The concentration of CPPU–BSA coated in the plate was diluted from 2.0 μg/mL to 0.25 μg /mL, and anti-CPPU mAb 14G1 was diluted in a series of two-fold dilutions from 1:2000. The optimal working concentration of the antibody 14G1 was defined as the dilution that resulted in an absorbance closest to 1.0. After optimization, the concentrations of CPPU–BSA and mAb 14G1 were 0.5 μg/mL and 0.016 μg/mL for icELISA (Figure S2a). Then, the process parameters of icELISA were optimized through single-factor experiments. The influence of blocking reagents commonly used in ELISA systems, OVA, skim milk, and BSA, were evaluated and compared for their effectiveness in reducing nonspecific binding. Finally, 5% skim milk was selected, which gave a minimum IC_50_ value (Figure S2b). CPPU is a lipophilic, small organic compound, and methanol has been reported to be a more acceptable co-solvent for CPPU during the immunochemical reaction [14]. The effect of methanol concentration on immunoassay performance was assessed. Since 5% methanol led to the lowest IC_50_ value of 0.0372 ng/mL, an optimal methanol concentration of 5% was selected for further investigation (Figure S2c). As shown in Figure S2d, when the ionic strength of the assay buffer was within the range of 0–544 mM, it had a significant impact on both IC_50_ and OD_max_. The ionic strength of 136 mM, which provided the lowest IC_50_ value, was selected. The pH value is one of the key factors influencing the characteristics of the assay. From Figure S2e, the value of pH 7.4 was selected as the optimum for the assay based on the favorable IC_50_ value. Under the optimal assay conditions, an indirect competitive ELISA standard curve was established (Figure 3). The IC_50_ and limit of detection (LOD, defined as the CPPU concentration corresponding to IC_10_) of icELISA were 0.0253 and 0.002 ng/mL, respectively. Moreover, the assay has a linear range (IC_20_–IC_80_) of 0.005–0.231 ng/mL.

### 3.3. Development and Optimization of MB-Based Assay

In order to improve the detection efficiency of classic icELISA for CPPU, MB-immobilized–CPPU–BSA was used to replace the step of coating the microplate with antigen, and HRP was directly labeled on mAb 14G1 to generate 14G1–HPR as a tracer, which can greatly simplify the detection procedure. Based on this, a one-step MB-based assay was established. The biotinylated CPPU–BSA was labeled on the streptavidin-modified MB by the biotin–avidin system to form MB–CPPU–BSA. The protein concentrations of the reaction solution before and after labeling were determined using BCA protein assay kit (Boster Biological Technology, Wuhan, China). Several biotinlyted CPPU–BSA amounts (0.5, 1.0, 2.0, 4.0, and 8.0 μg) were respectively mixed with 100 μg of SA-MB to optimize the amount of immobilized antigen. Anti-CPPU mAb–HRP was used to detect the immobilized antigen. As expected, absorbance values increased with increasing amounts of biotinylated CPPU–BSA (Figure S3a). The result indicated that 0.08 μg of biotinylated CPPU–BSA was saturatedly coupled to each microgram of MB. The successful immobilization of CPPU–BSA with MB was confirmed by transmission electron microscope (TEM) images, and the average diameter of the MB conjugates was 0.1 μm (Figure S3b). The HRP-conjugated mAb 14G1 was prepared according to an HRP labeling kit (Proteintech, Wuhan, China), and then 0.1 mg of anti-CPPU mAb 14G1 was chemically labeled with 0.1 mg of HRP to prepare 14G1–HRP, which served as a binder for recognizing the target antigen as well as a tracer to provide signal readout in the immunoassay.

To obtain better analytical performance of the MB-based assay, the reaction conditions were investigated. First of all, the working concentrations of MB–CPPU–BSA and mAb 14G1–HRP were determined by checkerboard titration as 0.1 mg/mL and 1:6400 dilution, respectively (Table S1). Then, the influence of other parameters on the assay was also optimized, in which the IC_50_ value was used as the main criterion. The effects of different ionic strengths and different pH values of the PBS solution on antigen–antibody binding in the MB-based assay were evaluated (Figure S4a,b). Based on slight variations in IC_50_ values, we found that ionic strength and pH had very limited negative effects on the MB-based assay, indicating that the MB-based assay was significantly more stable than traditional icELISA systems in the buffer solution with large fluctuations. The lowest IC_50_ values were observed in PBS with an ionic strength of 136 mM and a pH of 7.4, which were chosen for further optimization. Similarly, no significant variation was observed for IC_50_, as the final content of methanol did not exceed 10% (Figure S4c). The optimal methanol concentration was selected to be 5% for the lower IC_50_. To shorten the detection duration, the incubation time for the competitive reaction between MB–CPPU–BSA and free-CPPU for binding to mAb–HRP tracer was optimized in sequence. The effect of various lengths of competitive reaction time (10, 20, 30, and 40 min) on assay performance was also assessed (Figure S4d), and 20 min was found to be sufficient to reach a favorable IC_50_ value and maximum OD of around 1.0. Shorter incubation times may result in insufficient antigen–antibody binding, while longer incubation times not only further increased the maximum intensity but also led to higher background signals from non-specific binding. Therefore, incubation was stopped after 20 min, and beads were washed to remove unbound antibody.

Under the above optimal conditions, the standard inhibition curve of the MB-based assay was established as seen in Figure 3. The developed method afforded an IC_50_ of 0.0061 ng/mL and an LOD of 0.0004 ng/mL, along with the linear detection range (IC_20_–IC_80_) of 0.0016–0.0259 ng/mL. Compared with the classic icELISA based on mAb 14G1, the MB-based assay showed a 4.1-fold and 5-fold increase in sensitivity in terms of IC_50_ and LOD, respectively. The reason may be that the use of MBs as an immobile phase enables each reaction step to be homogeneous so that the antibody has more opportunities to bind to the target analytes in solution, thus improving the detection efficiency and sensitivity compared with microplate-based icELISA systems [31]. Moreover, the one-step strategy of the MB-based assay proved to be significantly simpler than the two-step icELISA, resulting in a reduction of operation time by almost three quarters.

Additionally, we have provided a detailed comparison of the analytical performance of these two developed methods with those of the previously documented immunoassay methods for CPPU detection, as shown in Table S2. The comparison results showed that the total analysis time of the developed MB-based assay was much less than other microplate-based enzyme immunoassays for CPPU [13,14,15,16], and they were even comparable to strip-based immunochromatography, which can complete the assay in 30 min [15]. Moreover, the IC_50_ and LOD values of the MB-based assay for CPPU were far lower than the reported immunoassays. Therefore, the MB-based assay established in this work showed great potential for rapid and sensitive detection of CPPU in practice.

### 3.4. Selectivity of MB-Based Assay and icELISA

To evaluate the selectivity of the proposed MB-based assay toward CPPU detection, five compounds (diuron, chlorotoluron, thidiazuron, linuron, and clofentezine) with similar structures of CPPU were selected to test the cross-reactivity (CR) of the mAb 14G1 of the construction method. In parallel, the selectivity of the icELISA was also assessed for comparison. As listed in Table 1, anti-CPPU mAb 14G1 showed extremely inconspicuous CRs with analogues in the MB-based assay and icELISA systems. When compared with previous studies [14,16], the ELISA-based antibodies exhibited obvious cross-reaction with thidiazuron. However, in the present study, an MB-based assay and icELISA-based mAb 14G1 had negligible cross-reaction with thidiazuron and other listed compounds, which exhibited better selectivity and could effectively guarantee the specific detection of CPPU.

### 3.5. Sample Analysis

**Figure 4:**
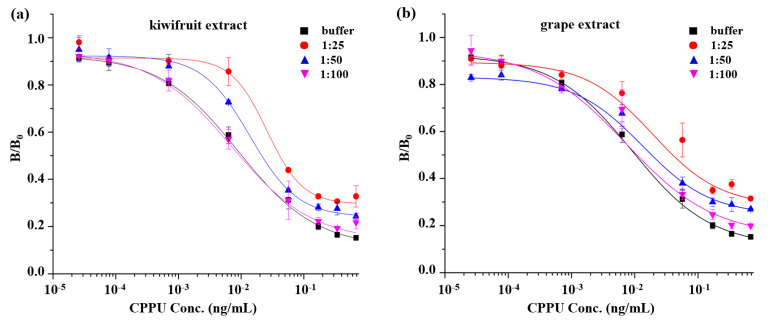
Matrix effects of kiwifruit and grape samples. Standard curves of MB-based assay for CPPU in assay buffer and diluted kiwifruit extracts (**a**) and grape extracts (**b**).

The food matrix effect is a key factor to address in immunoassays, as it may lower the color development or cause false positives by interfering with antigen–antibody binding. To minimize the interference of sample matrix on assay analysis, diluting the sample extracts is the simplest and most immediate way [32]. CPPU-free fruit (kiwifruit and grape) extracts were diluted and used to prepare a serial concentration of CPPU standards for the MB-based assay. After a series of dilutions of the sample extract, several matrix curves were created and then compared with the standard curve in assay buffer. As shown in Figure 4, the matrix interference from kiwifruit and grape was basically eliminated by a 100-fold dilution for the MB-based assay. Accordingly, considering the dilution factor of the sample, the resulting estimated LODs in kiwifruit and grape were 0.0191 μg/kg and 0.0168 μg/kg for the MB-based assay, respectively, which fulfilled the requirements of European Union regulatory standards for CPPU in various fruits (0.01 mg/kg).

A spike-and-recovery study was performed to evaluate the reliability of the MB-based assay in analyzing CPPU in the spiked fruit samples. Negative kiwifruit and grape samples were spiked with three different concentrations of CPPU (10, 20, and 50 μg/kg). The sample extractions were tested by the MB-based assay under optimal reaction conditions. As shown in Table 2, the average recoveries for the MB-based assay ranged from 86.0% to 120.0%, with the relative standard deviation (RSD) ranging from 1.7% to 10.0%, and for HPLC, the average recoveries ranged from 82.0% to 116.5%, with the RSD ranging from 2.5% to 13.8%. The above results indicated that the developed assay had the properties of accuracy and precision, and it could be a reliable analytical tool for quantitative detection of CPPU in actual samples.

## 4. Conclusions

In this work, we developed an anti-CPPU monoclonal antibody 14G1 with high affinity and great specificity, and we successfully applied this new antibody to establish a magnetic-bead-based analytical method for CPPU detection. The developed assay exhibited superiority in terms of sensitivity and rapidity compared with those of conventional icELISA. The detection limit of the MB-based assay was five times more sensitive than conventional icELISA. The analytical procedure of the MB-based assay was simplified because there were no preceding coating and blocking steps, and no secondary antibody was required. Moreover, the total assay duration of the MB-based assay was shortened to 35 min from several hours required in conventional icELISA. Furthermore, we also verified the satisfactory performance of the established MB-based assay by analyzing spiked samples. Therefore, the proposed MB-based assay could be considered a feasible quantitative method for CPPU analysis without expensive equipment, and it allows sensitive and rapid screening of CPPU in food. This work explored the analysis of CPPU by an MB-based immunoassay and provided a basis for promoting the application of more immunosensors in the analysis of other agrochemicals. The principle of this assay can be transferred to other formats (e.g., electrochemical), and it may replace ELISA as a promising alternative immunochemical method.

## Figures and Tables

**Table 1:** Cross-reactivity (CR) of MB-based assay and icELISA with CPPU analogues.

Analytes	Structure	MB-Based Assay		icELISA	
		IC_50_ (ng/mL)	CR (%)	IC_50_ (ng/mL)	CR (%)
Forchlorfenuron (CPPU)	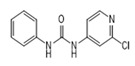	0.0061	100	0.0253	100
Diuron	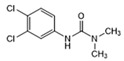	>1.0	<0.6	>2.5	<1.0
Chlorotoluron	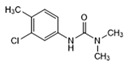	>1.0	<0.6	>2.5	<1.0
Thidiazuron	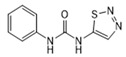	>1.0	<0.6	>2.5	<1.0
Linuron	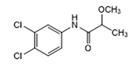	>1.0	<0.6	>2.5	<1.0
Clofentezine	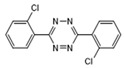	>1.0	<0.6	>2.5	<1.0

**Table 2:** Recoveries of CPPU from spiked samples by MB-based assay and HPLC (*n* = 3).

Samples	Spiked Level (μg/kg)	MB-Based Assay			HPLC		
		Mean ± SD (μg/kg)	Recovery (%)	RSD (%)	Mean ± SD (μg/kg)	Recovery (%)	RSD (%)
Kiwifruit	10	8.6 ± 0.6	86.0	7.0	8.9 ± 0.5	89.0	5.6
	20	19.3 ± 0.5	96.5	2.6	23.3 ± 3.0	116.5	12.9
	50	45.1 ± 4.5	90.2	10.0	52.3 ± 1.3	104.6	2.5
Grape	10	12.0 ± 0.2	120.0	1.7	8.7 ± 1.2	87.0	13.8
	20	20.3 ± 1.2	101.5	5.9	16.4 ± 0.5	82.0	3.0
	50	46.5 ± 1.3	93.0	2.8	48.4 ± 3.6	96.8	7.4

## Data Availability

Not applicable.

## Supplementary Materials

The following supporting information can be downloaded at: https://www.mdpi.com/article/10.3390/bios13060593/s1, Figure S1. Synthesis route of hapten CPPU-COOH; Figure S2. Optimization of the operating conditions of icELISA. (a) coating antigen and mAb 14G1 concentration, (b) blocking reagent, (c) methanol concentration, (d) ionic strength in PBS buffer, and (e) pH of buffer; Figure S3. (a) Optimization of the amounts of biotinylated CPPU–BSA conjugated to the streptavidin-modified magnetic beads and (b) characterization of MB–CPPU–BSA conjugate by transmission electron microscope (TEM) image; Figure S4. Optimization of the operating conditions of MB-based assay. (a) Ionic strength in PBS buffer, (b) pH of buffer, (c) methanol concentration, and (d) competitive reaction time; Table S1. Optimization of concentrations of mAb–HRP and MB–CPPU–BSA for MB-based assay; Table S2. Analytical performance of the reported immunoassays for CPPU detection.
