# Supplementary material for: A Magnetic-Bead-Based Immunoassay with a Newly Developed Monoclonal Antibody for Rapid and Highly Sensitive Detection of Forchlorfenuron
Source: Biosensors (Basel). 2023 May 30;13(6):593. doi: 10.3390/bios13060593 (PMC10296238; doi:10.3390/bios13060593)
Supplement: Supplementary file 1 [file biosensors-13-00593-s001.zip › biosensors-2355811-supplementary.pdf]

Supporting Information

A Magnetic-Bead-Based Immunoassay with a Newly Developed Monoclonal Antibody for Rapid and Highly Sensitive Detection of Forchlorfenuron

Yubao Shan [1,2], Ting He [1,*], Ying Li [1,3], Jiang Zhu [1,3], Xiali Yue [2] and Yunhuang Yang [1,3,4,*]

[1] State Key Laboratory of Magnetic Resonance and Atomic Molecular Physics, Key Laboratory of Magnetic Resonance in Biological Systems, National Center for Magnetic Resonance in Wuhan, Wuhan Institute of Physics and Mathematics, Innovation Academy for Precision Measurement Science and Technology, Chinese Academy of Sciences—Wuhan National Laboratory for Optoelectronics, Wuhan 430071, China; shanyubao@wipm.ac.cn (Y.S.); liying@apm.ac.cn (Y.L.); jiangzhu@apm.ac.cn (J.Z.)
[2] Department of Chemistry, College of Science, Huazhong Agricultural University, Wuhan 430070, China; yxl@mail.hzau.edu.cn
[3] University of Chinese Academy of Sciences, Beijing 100049, China
[4] Optics Valley Laboratory, Wuhan 430074, China
* Correspondence: ht210@apm.ac.cn (T.H.); yang_yh@apm.ac.cn (Y.Y.); Tel.: +86-27-87198006 (T.H.); +86-27-87199541 (Y.Y.)

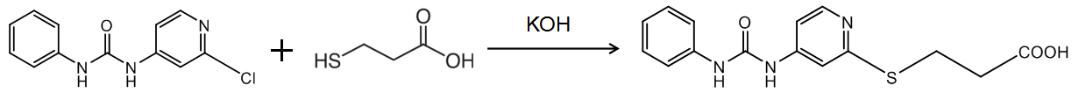

Figure S1. Synthesis route of hapten CPPU-COOH. CPPU was coupled with 3-mercaptopropionic acid, and an alkyl mercaptoacid was introduced on the pyridine ring of CPPU.

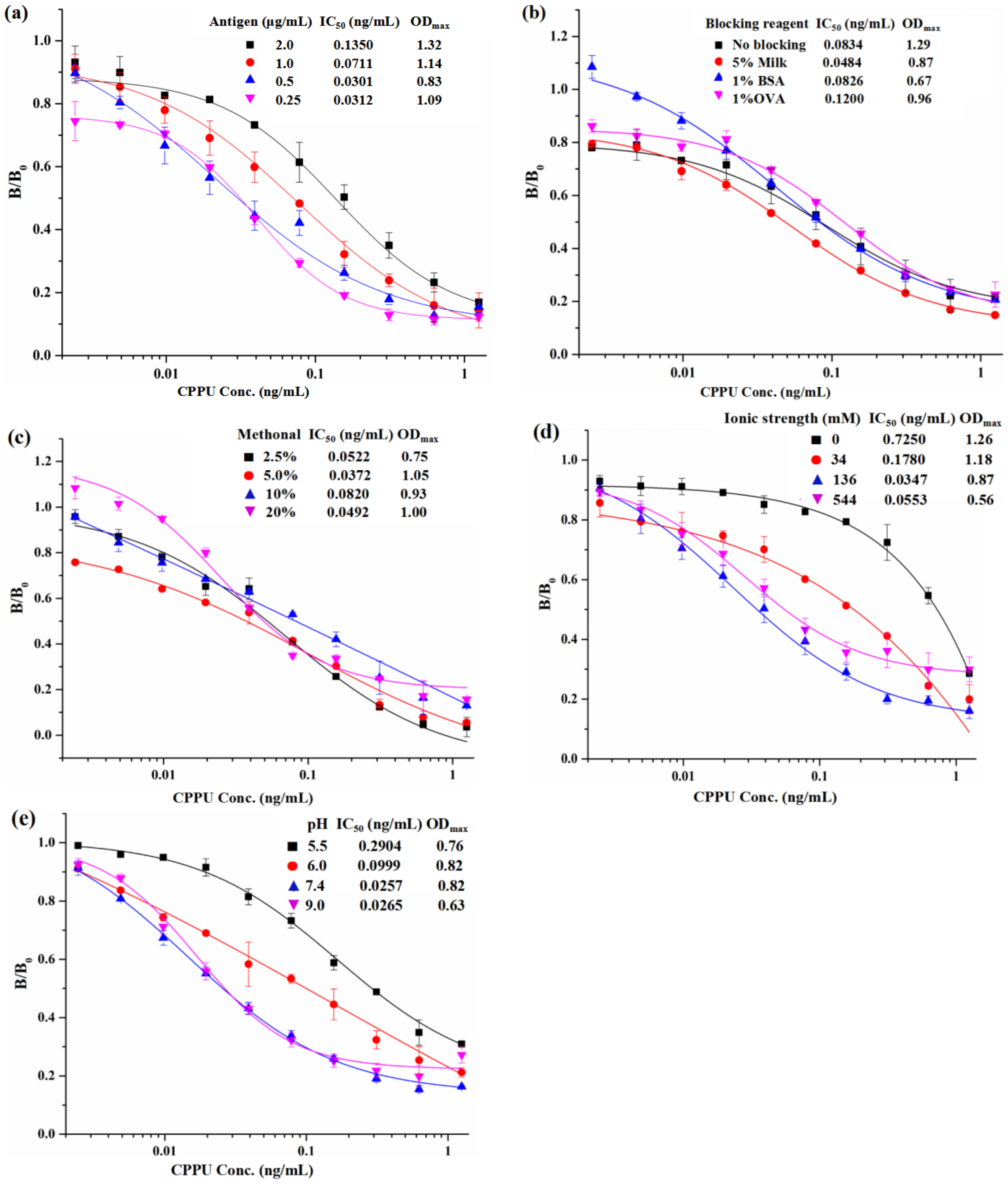

Figure S2. Optimization of the operating conditions of icELISA. (a) coating antigen and mAb 14G1 concentration, (b) blocking reagent, (c) methanol concentration, (d) ionic strength in PBS buffer, and (e) pH of buffer.

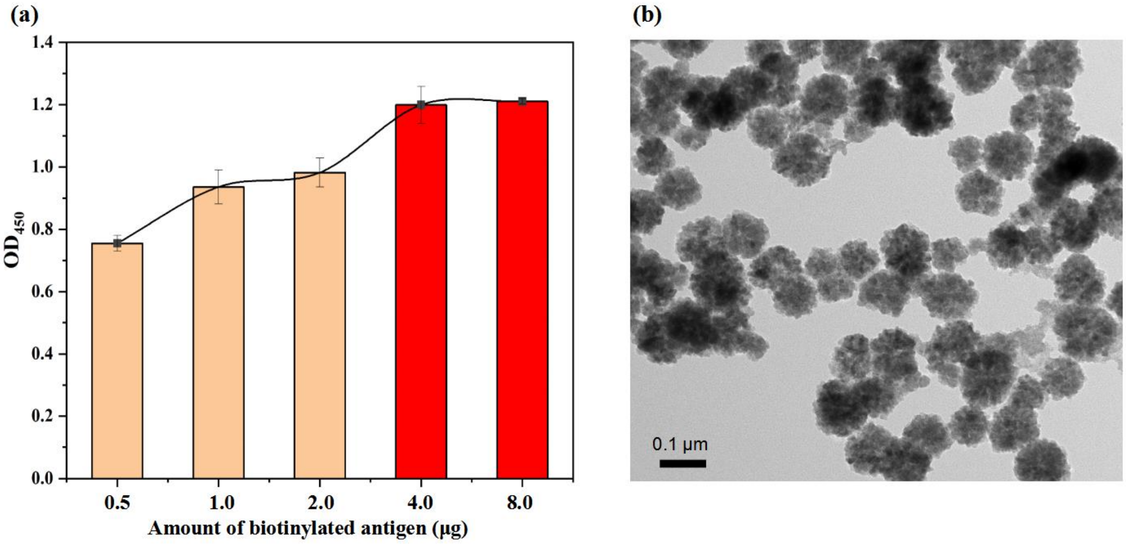

Figure S3. (a) Optimization of the amounts of biotinylated CPPU-BSA conjugated to the streptavidin-modified magnetic beads, and (b) characterization of MB-CPPU-BSA conjugate by transmission electron microscope (TEM) image.

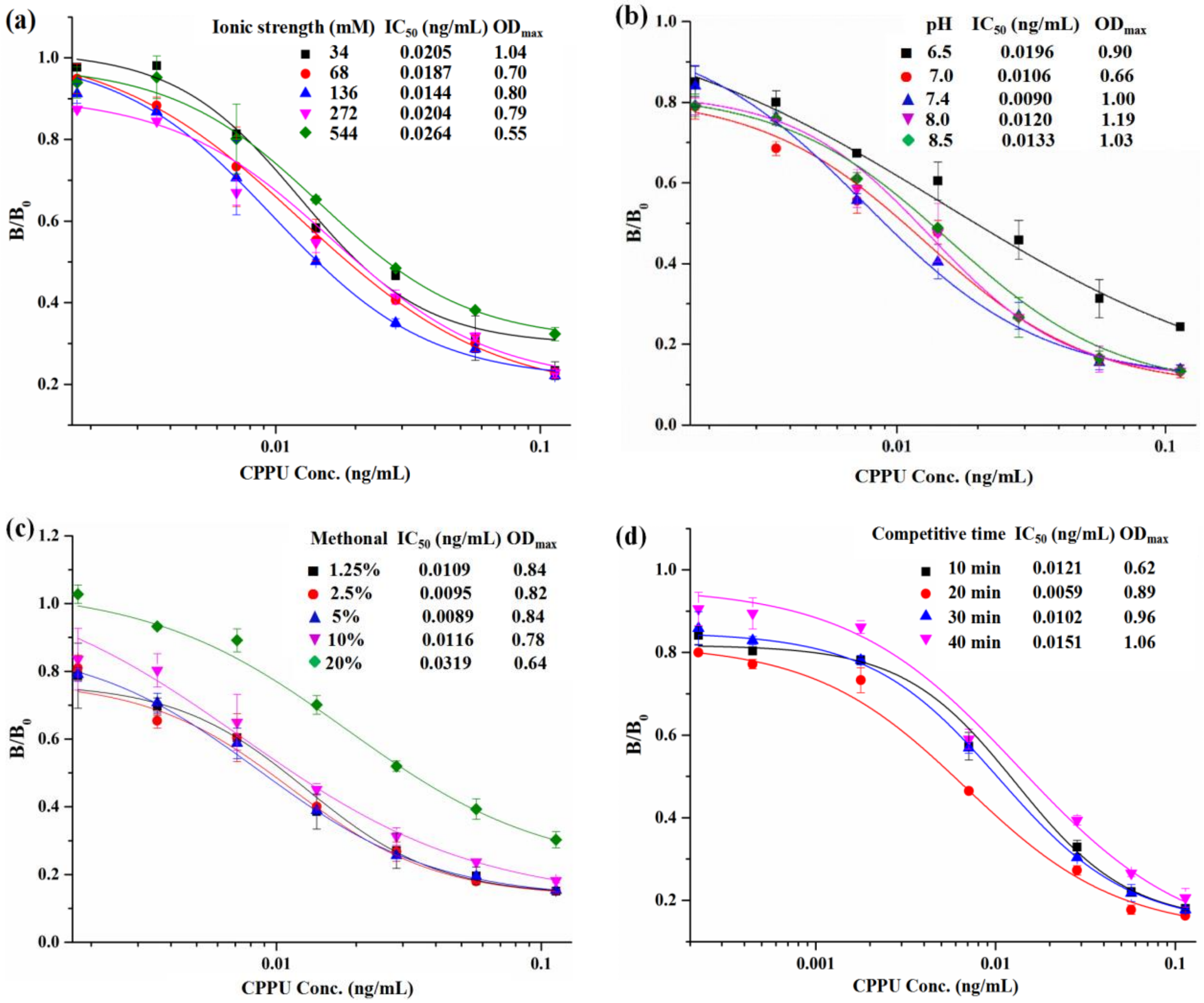

Figure S4. Optimization of the operating conditions of MB-based assay. (a) ionic strength in PBS buffer, (b) pH of buffer, (c) methanol concentration, and (d) competitive reaction time.

Table S1. Optimization of concentrations of mAb-HRP and MB-CPPU-BSA for MB-based assay.

**Dilution factor of mAb-HRP**	**MB-CPPU-BSA concentration (μg/μL)**	**IC_{50} (ng/mL)**	**OD_{max}**
1:400	0.0125	0.0315	0.921
1:800	0.025	0.0147	0.863
1:1600	0.05	0.0139	0.812
1:3200	0.10	**0.0132**	0.921
1:6400	0.20	0.0140	0.833

Table S2. Analytical performance of the reported immunoassays for CPPU detection.

**Method**	**Recognition reagent**	**IC_{50} (ng/mL)**	**LOD (ng/mL)**	**Linear range (ng/mL)**	**Assay time**	**Reference**
dcELISA	pAb	0.077	0.012	0.025-0.276	70 min	[13]
dcELISA	mAb	0.063	0.01	-[a]	70 min	[14]
LFIA[b]	mAb	0.286	0.045	0.089-0.916	30 min	[15]
icELISA	mAb	0.05	0.013	0.022-0.156	130 min	[15]
icELISA	mAb	1.04	0.16	0.31-3.43	80 min	[16]
MB-based assay	mAb	0.0061	0.0004	0.0016-0.0259	35 min	This work
icELISA	mAb	0.0253	0.002	0.005-0.231	135 min	This work

Notes: [a] Data not provided; [b] LFIA means lateral flow immunoassay.

References

[13] Suarez-Pantaleon, C.; Mercader, J.V.; Agullo, C.; Abad-Somovilla, A.; Abad-Fuentes, A. Hapten synthesis and polyclonal antibody-based immunoassay development for the analysis of forchlorfenuron in kiwifruit. *J. Agric. Food Chem.* **2010**, *58*, 8502–8511.

[14] Suarez-Pantaleon, C.; Esteve-Turrillas, F.A.; Mercader, J.V.; Agullo, C.; Abad-Somovilla, A.; Abad-Fuentes, A. Development and validation of a direct competitive monoclonal antibody-based immunoassay for the sensitive and selective analysis of the phytoregulator forchlorfenuron. *Anal. Bioanal. Chem.* **2012,** *403*, 2019–2026.

[15] Suarez-Pantaleon, C.; Wichers, J.; Abad-Somovilla, A.; van Amerongen, A.; Abad-Fuentes, A. Development of an immunochromatographic assay based on carbon nanoparticles for the determination of the phytoregulator forchlorfenuron. *Biosens. Bioelectron.* **2013**, *42*, 170–176.

[16] Liu, X.M.; Xie, B.; Cheng, Y.J.; Luo, L.; Liang, Y.F.; Xiao, Z.L. A sensitive monoclonal-antibody-based ELISA for forchlorfenuron residue analysis in food samples. *Biosensors-Basel* **2022**, *12*, 78.
